# Ultrasonography findings in knee osteoarthritis: a prospective observational cross-sectional study of 100 patients

**DOI:** 10.1038/s41598-021-95419-3

**Published:** 2021-08-16

**Authors:** Claudia Andreia Rabay Pimentel Abicalaf, Leticia Naomi Nakada, Felipe Ricardo Aquino dos Santos, Ichiro Akiho, Artur Cesar Aquino dos Santos, Marta Imamura, Linamara Rizzo Battistella

**Affiliations:** 1 Instituto de Medicina Fisica e Reabilitacao, Hospital das Clinicas HCFMUSP, Faculdade de Medicina, Universidade de Sao Paulo, São Paulo, SP Brazil; 2 Faculdade de Medicina FMUSP, Universidade de Sao Paulo, São Paulo, SP Brazil

**Keywords:** Musculoskeletal system, Osteoarthritis

## Abstract

Worldwide, knee osteoarthritis (KOA) accounts for 2.2% of total years lived with disability. There is a low correlation between joint tissue damage and pain intensity. Periarticular structures may be involved and cannot be identified in X-rays. To describe the main ultrasonography (USG) changes in symptomatic patients with primary KOA; to correlate the number of USG findings with KOA severity assessed by Kellgren and Lawrence (K&L) radiological scores, with pain intensity measured by a visual analogue scale (VAS) and with functioning scores assessed with the Timed up and go test (TUG) and Western Ontario and McMaster Universities (WOMAC) questionnaire. 100 patients with primary symptomatic KOA were assessed with X-ray and USG. Quantitative and qualitative analyses were evaluated in a systematic manner. The most frequent findings were joint effusion, pes anserinus bursitis, quadriceps tendon enthesopathy, popliteal cyst, iliotibial band tendinitis and patellar tendinitis. Pearson’s correlation analysis demonstrated a significant moderate positive association between VAS scores and the number of USG findings (r = 0.36; p < 0.0001). The number of USG findings was different between K&L grades I and III (p = 0.041), I and IV (p < 0.001), and II and IV (p = 0.001, analysis of variance with Bonferroni correction). There was significant association between number of USG findings and TUG (r = 0.18; p = 0.014) and WOMAC scores for pain (r = 0.16; p < 0.029) and physical function domains (r = 0.16; p < 0.028). The most frequent USG finding was joint effusion. Periarticular structures should be explored as potential sources of pain and disability.

## Introduction

Musculoskeletal disorders are the group that most contribute to the need for rehabilitation, due to their high prevalence and contribution to total years lived with disability^1^. Among these disorders, knee osteoarthritis (KOA) is a leading cause of disability in the elderly population and a common health condition in this population^2^. Worldwide, KOA is the 11th leading cause of global disability, accounting for 2.2% of total years lived with disability^2^. With the aging of the global population, it is relevant to keep people healthy and functioning properly. Currently, there are no disease-modifying interventions for KOA, despite the understanding of the joint pathology at the molecular level^3,4^. In fact, there is a low correlation between joint tissue damage and pain intensity^5–7^. Severe joint damage visualized in the knee X-ray is present in asymptomatic people with KOA^5^. On the other hand, 10% of people with severe knee pain have normal X-rays^6^. About 10–20% of people with KOA develop generalized pain^7^. Lack of proper understanding of the mechanisms involved in pain generation, maintenance and amplification and therefore adequate management strategies leads to an exponential increase in the number of total knee replacements worldwide^8^.

Several authors have already identified the presence of periarticular (tendons, ligaments and bursae) or intra-articular changes in people with symptomatic KOA^3^. The main changes identified in a knee ultrasonography (USG) assessment revealed joint effusion, popliteal cysts and tendinopathies^9–15^. USG is a non-invasive, fast and operator-dependent imaging technique that allows the visualization of changes in these structures. However, there is remaining controversy about the existence of correlations between USG findings, Kellgren and Lawrence (K&L) grades^14^, pain scores and functionality in previous studies^10,11,13,15^.

Therefore, the objectives of our study were to describe the main USG changes in symptomatic patients with primary KOA; to correlate the number of USG changes and the severity of the X-ray findings using the K&L radiological scores; and to determine if there is a significant correlation between the number of the USG changes and the pain intensity measured by a visual analogue scale (VAS) and functioning scores assessed with Timed up and go test (TUG) and Western Ontario and McMaster Universities Osteoarthritis Index (WOMAC) questionnaires.

## Patients and methods

### Patients

This is a prospective observational cross-sectional study without a control group. We assessed the knee USG findings in persons with primary symptomatic KOA. Data from this study were obtained by another study entitled “Inhibitory deficit as a marker of neuroplasticity in rehabilitation” approved by Hospital das Clinicas Comite de Analise de Projetos de Pesquisa CAPPesq, by the local Ethical Committee CAPPesq (CAAE: 86832518.7.0000.0068). A total of 100 consecutive consenting patients with primary symptomatic KOA (194 knees) were included following these criteria: age > 50 years; clinical and radiological diagnosis of KOA^16^; high or moderate pain intensity: VAS > 4; knee pain duration > 3 months; absence of severe psychiatric disorder requiring follow-up assistance; no clinical diagnosis of associated fibromyalgia; absence of associated systemic inflammatory rheumatic diseases; absence of a history of neoplasms and absence of predominant clinical manifestations in other joints. Written informed consent was obtained from all the participants.

### Methods

All methods were carried out in accordance with relevant guidelines and regulations.

#### Clinical evaluation

We excluded secondary causes of KOA during the clinical evaluation of all patients. All patients were evaluated for pain using a VAS^17^ and knee functioning using the WOMAC scores^18^ and the TUG^19^.

#### Radiographic evaluation

**Figure 1:**
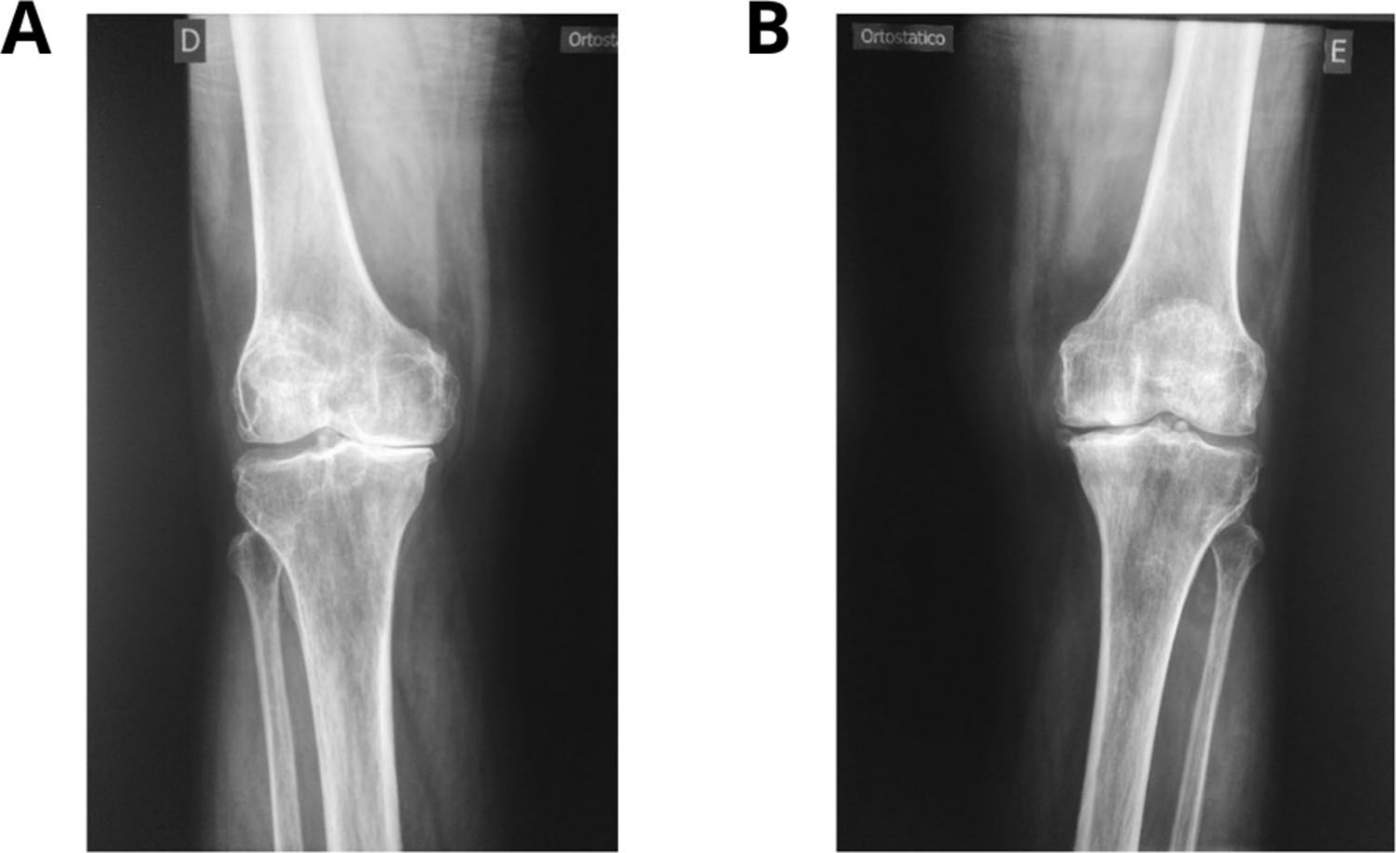
Knee X-ray. (**A**) Right knee, osteoarthritis Kellgren & Lawrence grade IV. (**B**) Left knee, osteoarthritis Kellgren & Lawrence grade III.

A Luminus RF X-ray equipment (Siemens, Germany) was used for the knee X-ray exams. Plain radiographic knee examinations were performed in all patients in three incidences: anteroposterior (AP) with load, lateral and axial patella. Classification of KOA severity was performed using the K&L scores^20^ (Fig. 1) assessed with regards to bone limits such as anatomical repairs, muscle–tendon changes, insertions and enthesis. The same radiologist analyzed all X-ray exams.

#### Ultrasonographic evaluation

Knee USG evaluations were performed using a Siemens Sonoline G40 USG equipment with multi-frequency linear transducers of 5–13 MHz (Siemens, Germany). Patients lied down in the dorsal decubitus with the examined knee flexed at a 30-degree angle using a cushion device. Images were obtained in the axial plane (transversal) to assess the patellar region. In the sequence, we analyzed, in the longitudinal plane, the enthesis with insertion of the quadriceps tendon, the patellar tendon (proximal and distal), as well as the medial and lateral collateral ligaments, the topography of the iliotibial tract and the components of the pes anserinus bursae. Subsequently, patients were placed in ventral decubitus where the axial and longitudinal planes allowed the visualization of the popliteal fossa. In this view, we were able to assess the presence of effusion as well as of popliteal cyst, also known as Baker’s cyst.

Quantitative and qualitative analyses (changes of the echogenicity of the muscles and tendons, presence or absence of peri-tendinous fluid/synovial thickening and/or joint fluid [effusion] as well as presence or absence of cysts, especially in the popliteal fossa) were all assessed in a systematic manner.

##### Definition of the main USG findings

**Figure 2:**
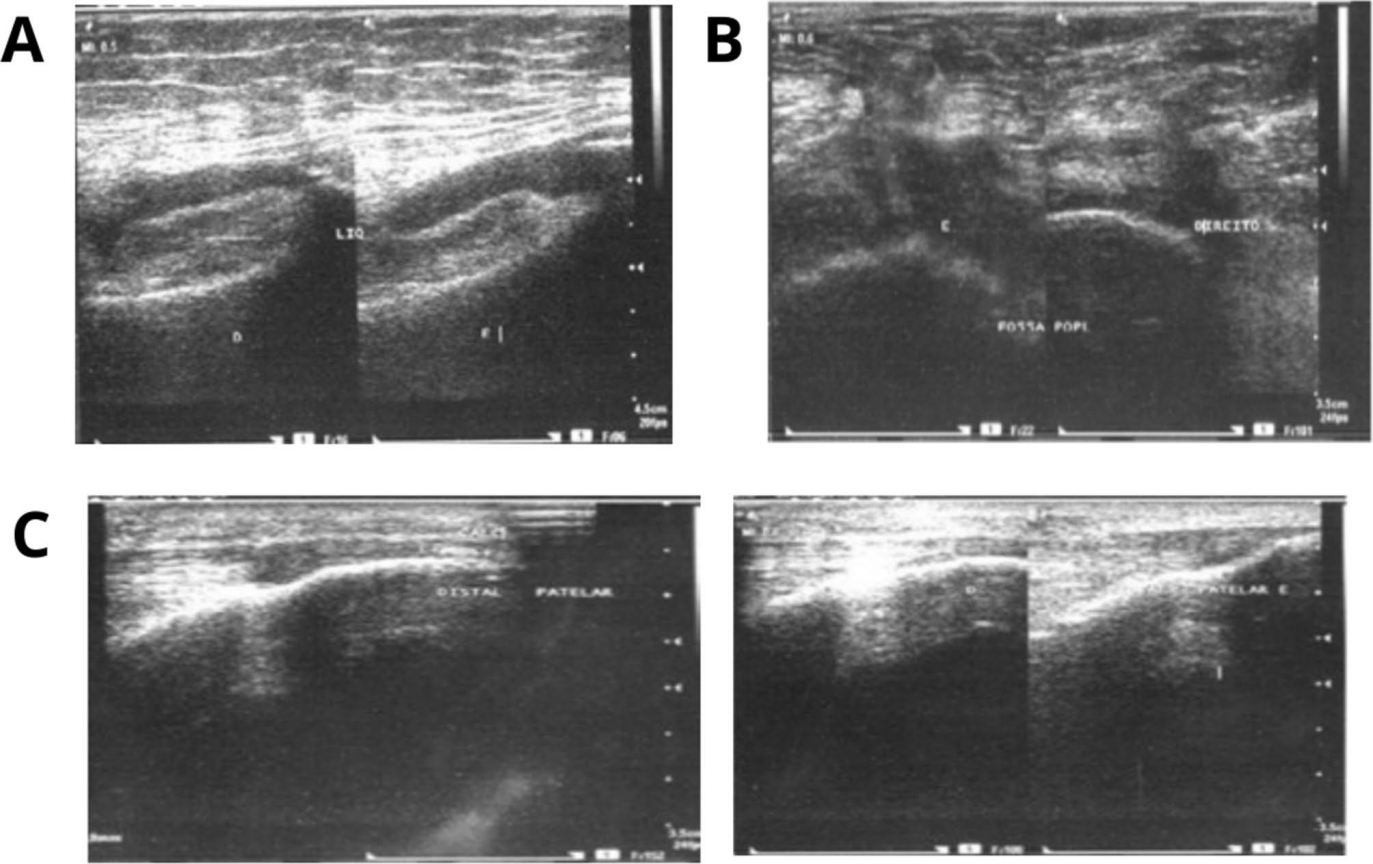
Knee ultrasonography. (**A**) Joint effusion. (**B**) Popliteal cyst. (**C**) Patellar tendinitis.

*Effusion* the presence of increased articular fluid (> 4 mm), visualized as a hypoechogenic or anechogenic displaceable material in the knee joint cavity (Fig. 2A).

*Popliteal cyst* anechogenic and with clear boundaries cystic formation situated between medial gastrocnemius and semimembranosus muscles (Fig. 2B). It is assessed in three different axes: longitudinal, axial and anteroposterior.

*Pes anserinus bursitis* thickened and hypoechogenic structure found in pes anserinus distal portion, on leg’s medial side, in a longitudinal approach.

*Suprapatellar tendinitis* thickened and hypoechogenic distal part of the quadriceps muscle, observed in its larger axis (Fig. 2C).

All USG examinations were performed using the same equipment, the same methodology and by the same two experienced examiners. All ultrasound examinations followed a rigorous execution criterion, performed by two experienced examiners, standardizing a sequenced ultrasound parameters analysis.

#### Statistical analysis

Ultrasound findings of the knees with radiological diagnosis of osteoarthritis (K&L from I to IV) were quantified. Then, pain intensity measured by the VAS for each knee was tested in association with the number of ultrasound findings by Pearson correlation. Knee function, assessed by the TUG and the WOMAC in the pain, stiffness and difficulty to perform exercises domains, were also tested in association with the number of USG findings using the Pearson’s correlation (r). The number of knees included in the evaluations was 194, a sample size that allowed the assumption of the central limit theorem, i.e. this sample has an approximately normal distribution^21,22^.

In a secondary analysis, we tested whether the number of USG findings varies significantly according to the K&L classification using the analysis of variance (ANOVA) with Bonferroni correction for multiple analysis.

Demographic data regarding age, gender, time of pain, body mass index (BMI) and education were analyzed as to whether they were also associated with the mean baseline pain intensity between the two knees, K&L scores ≥ 1, for a better description of demographic influence on pain.

Age, time of pain, and BMI were analyzed for pain intensity by simple linear regression, gender was analyzed by Student’s t-test, and education (illiterate, elementary, middle, and higher) by variance analysis. Demographic data were not tested in conjunction with ultrasound changes because they were not related to just one knee.

Statistical significance was considered if p < 0.05, two-tail, and analysis was performed using the Stata 14^®^ statistical package.

### Ethics approval and consent to participate

Approved by Hospital das Clinicas Comite de Analise de Projetos de Pesquisa CAPPesq. Already approved by the local Ethical Committee CAPPesq (CAAE: 86832518.7.0000.0068).

## Results

One hundred patients diagnosed with symptomatic KOA were included in the study. Six patients had unilateral total knee arthroplasty; therefore, 194 knees were evaluated. A single USG finding was observed in 48 knees (24.74%), two in 47 (24.23%), three in 26 (13.40%), four in 24 knees (12.37%) and five in 27 knees (13.92%). USG findings were not observed in 22 knees (11.34%; 18 patients). The most frequent findings were effusion (n = 150; 77.32%), pes anserinus bursitis (n = 84; 43.30%), popliteal cyst (n = 57; 29.38%), enthesopathy of quadriceps tendon (n = 58; 29.9%), iliotibial band tendinitis (n = 49; 25.26%), and patellar tendinitis (n = 30; 15.46%). The demographic description of the patients included in this study is presented in Table 1. 22 knees from 12 patients had no USG findings. 4 patients did not have USG findings bilaterally (8 knees), and 14 patients did not have them unilaterally (14 knees). Their characteristics are shown in Table 2.

**Table 1:** Demographics.

Population, n	100
Knees, n	194
Age (years), mean (SD)	68.90 (9.74)
Male, n (%)	18 (18.00)
Female, n (%)	82 (82.00)
BMI (kg/m^2^), mean (SD)	31.89 (5.33)
Duration of pain (years), mean (SD)	7.50 (8.27)
**Kellgren and Lawrence grade, n (%)**	
0	1 (0.52)
I	58 (29.90)
II	48 (24.74)
III	35 (18.04)
IV	52 (26.80)
VAS, mean (SD)	5.60 (2.81)
WOMAC pain, mean (SD)	10.98 (3.94)
WOMAC stiffness, mean (SD)	4.68 (1.95)
WOMAC difficulty, mean (SD)	35.97 (13.67)
TUG, mean (SD)	15.86 (8.0)
No USG finding, n (%)	22 (11.34)
Single USG finding, n (%)	48 (24.74)
Two USG findings, n (%)	47 (24.23)
Three USG findings, n (%)	26 (13.40)
Four USG findings, n (%)	24 (12.37)
Five or more USG findings, n (%)	27 (13.92)
**Ultrasonography findings**	
Effusion, n (%)	150 (77.32)
Pes anserinus bursitis, n (%)	84 (43.30)
Enthesopathy of quadriceps tendon, n (%)	58 (29.9)
Popliteal cyst, n (%)	57 (29.38)
Iliotibial band tendinites, n (%)	49 (25.26)
Patellar tendinitis, n (%)	30 (15.46)

*n* number, *%* percentage, *SD* standard deviation, *BMI* body mass index, *kg/m*^*2*^ kilograms per meter^2^, *VAS* visual analogue scale, *USG* ultrasonography, *WOMAC* Western Ontario and McMaster Universities Osteoarthritis Index, *TUG* timed up and go test.

**Table 2:** Clinical and radiographic data of knees with no USG finding.

Population, n	12
Knees, n	22
Age (years), mean (SD)	67.28 (8.76)
Duration of pain (years), mean (SD)	7.05 (9.17)
**Kellgren and Lawrence grade, n (%)**	
0	0 (0)
I	9 (40.91)
II	9 (40.91)
III	4 (18.18)
IV	0 (0)
VAS, mean (SD)	3.69 (3.22)
WOMAC pain, mean (SD)	10.33 (4.51)
WOMAC stiffness, mean (SD)	4.90 (1.75)
WOMAC difficulty, mean (SD)	32.76 (17.70)
TUG, mean (SD)	14.55 (5.15)

*USG* ultrasonography, *n* number, *%* percentage, *SD* standard deviation, *VAS* visual analogue scale, *WOMAC* Western Ontario and McMaster Universities Osteoarthritis Index, *TUG* timed up and go test.

When categorizing the sample by K&L grades, we observed that the number of USG findings was different for classifications I and III (p = 0.041), I and IV (p < 0.001) and for classifications II and IV (p < 0.001), ANOVA with Bonferroni correction. In all other comparisons in the K&L classifications, there was no evidence of a different number of USG findings.

Demographically, the only characteristic that correlated with pain intensity was the education level; higher education levels correlated with less pain reported by the patients. Neither sex, age, pain duration nor BMI demonstrated significant correlations. Post hoc analysis investigated a possible correlation between the number of USG findings and the education level, suggesting collinearity and therefore education as a confounder for the analysis. It was not possible to demonstrate this event as Pearson’s correlation (r) between the variables was low, and without a statistical significance (r = 0.03, p = 0.658). None of the other demographic variables correlated with pain. The results are presented in Table 3.

**Table 3:** Results.

	Statistics	p value
Knee ultrasound findings and pain intensity	0.36*	< 0.0001
Knee ultrasound findings and K&L classification, mean findings (SD)	K&L I: 1.74 (1.45)	< 0.0001**
	K&L II: 1.77 (1.31)	
	K&L III: 2.6 (1.59)	
	K&L IV: 3.4 (1.54)	
Knee ultrasound findings and TUG	0.18*	0.014
Knee ultrasound findings and WOMAC Pain	0.16*	0.029
Knee ultrasound findings and WOMAC Rigidity	0.07*	0.337
Knee ultrasound findings and WOMAC physical difficulties	0.16*	0.028
Age	0.11*	0.110
Pain duration (months)	0.07*	0.352
BMI	0.06*	0.411
Gender: “n of knees” and pain mean	F: n = 164; 5.64 (2.78)	0.338^†^
	M: n = 34; 5.13 (2.97)	
Education: n, mean pain intensity by K&L grade (SD)	Illiterate: n = 4; 9.77 (0.17)	0.0002***
	Elementary: n = 79; 6.16 (2.68)	
	High-School, n = 62; 5.35 (2.76)	
	Superior, n = 52; 4.55; (2.73)	

*n* number, *SD* standard deviation, *BMI* body mass index, *F* female, *M* male, *K&L* Kellgren and Lawrence, *WOMAC* Western Ontario and McMaster Universities Osteoarthritis Index, *TUG* timed up and go test.

*Pearson Correlation (r).

**ANOVA of number of USG findings for K&L I and K&L III, p = 0.041, K&L I and K&L IV, p < 0.001, K&L II and K&L IV, p < 0.001 (with Bonferroni correction); [AS5].

***ANOVA education illiterate and superior, p = 0.002, illiterate and high school, p = 0.01; and fundamental and superior, p = 0.006 (with Bonferroni correction).

^†^Unpaired student t-test.

## Discussion

Our study identified USG changes in 78% of the examined knees. Main USG findings were small to moderate joint effusion, pes anserinus bursitis, quadriceps enthesopathy, popliteal cyst, iliotibial band tendinitis and patellar tendinitis. Together with the high prevalence of USG findings, we identified a significant positive correlation between the number of USG findings and pain intensity scores measured by a VAS.

Our findings are similar to the observations of several other authors^9,12,13,15,23,24^. Mendieta et al*.*^9^ found significant positive correlation between the presence of pain and greater K&L grades. The presence of Baker’s cyst tended to be statistically more present in patients with KOA compared with the control group (p = 0.06) and, when present, the odds ratio for pain was 5.5 (95% Confidence Interval 1 to 31.05). Hall et al*.*^12^ identified a positive association of the findings of joint effusion with higher radiographic scores (Nottingham logically derived Line Drawing Atlas), and of joint effusion with pain intensity. In the study of Malas et al*.*^13^, 122 knees from 61 patients were classified into two groups based on symptom severity. They observed that the presence of Baker’s cyst and joint effusion seen with USG was more prevalent in the group of more symptomatic knees when compared with the group of less symptomatic ones, such that the authors suggest that Baker’s cyst and joint effusion are associated with pain and function. Abd El Monaem et al.^15^ reported a positive association between pain intensity and USG detected joint effusion and Baker’s cyst volumes, as well as a correlation between functionality scores measured by WOMAC and USG features (joint effusion and Baker’s cyst volumes and thickness of the quadriceps tendon). In the study of Monteforte et al.^23^, joint effusion and Baker’s cyst were also the main USG findings described in patients with symptomatic KOA. They detected a statistically significant relevance for joint effusion and presence of Baker’s cyst in the symptomatic group. Naredo et al.^24^ reported that the presence of joint effusion was associated to the presence of Baker’s cyst and to higher pain intensity measured by VAS. Also, they found that pain intensity was not related to the BMI.

Even though Bevers et al.^11^ identified the presence of joint effusion and Baker’s cyst as frequent findings, differently from our study, they were not able to demonstrate any associations between different USG features and the level of knee pain, assessed with a numerical rating scale and with the Knee Injury and Osteoarthritis Outcome Score. The authors alluded that a possible explanation of their controversial findings compared with the previous USG features described in the literature was due to selection bias. Their study included mainly patients with less severe KOA (K&L grades I and II) and people with relatively high levels of pain (mean numerical rating scale score = 6.1; mean Knee Injury and Osteoarthritis Outcome Score = 57.0). Because of assessing a more heterogeneous sample than the one we did, Bevers et al., claim that their findings may be more accurate than the previous smaller trials already published, with much larger variability of KOA severity and intensity of pain. Our sample size, on the other hand, was also heterogeneous as in the previous publications. Other possible explanation for the disagreement in the results may derive from the USG machines and probes used in the different studies. Our high-frequency transducer together with experienced sonographers and a systematic assessment protocol with proper patient positioning allowed the visualization of all predefined anatomical structures and possible imaging changes.

USG is radiation-free, non-invasive, simple to be used, practical and a low-cost and non-time-consuming evaluation tool. The inter-observer agreement is excellent for Baker’s cyst (k-value: 0.85) using USG in KOA^25^. The intra-observer agreement is also higher for junior sonographers compared with beginners^26^. In our study, two experienced sonographers performed all USG evaluations.

We fully agree with the comments by Iagnocco et al.^26^ that USG is a reliable assessment tool for several anatomical structures that may be involved in KOA. Early diagnosis of such anatomical changes may guide the introduction of specific interventions to address these findings. Retrospective review of sonographic images evidenced a 100% sensitivity, specificity, positive predictive value, negative predictive value, and accuracy in the diagnosis of Baker’s cyst when hypoechoic or anechoic fluid was present between the semimembranosus and medial gastrocnemius tendons^25^. Compared to magnetic resonance imaging (MRI), USG identified 100% (12/12) of joint effusions and 100% (5/5) of Baker’s cysts^26^. USG reliably demonstrated joint effusions and Baker’s cysts^27^. Also, differently from the MRI, USG allows the dynamic evaluation of tendons and liquid mobilization.

Joint effusions, on the other hand, demonstrated relatively low intra- and inter-observer reliability^28^. There is no validated protocol to measure effusion using USG^29^. However, examining the patients with the knees flexed at 30 degrees allow the detection of small effusion compared to the examination with extended knees^30^. We identified joint effusion in 77.32% of our patients. A possible explanation for this high frequency is that patients with KOA tend to present a lack of proprioception^31^. Joint effusion and consequently distended capsule may be a compensatory mechanism in the attempt to increase proprioception. Then, the high percentage of this finding may suggest the need to recover proprioceptive functioning.

We have systematically visualized the anserine bursa, the patellar tendon and the iliotibial band in all of our patients. Interestingly, we have identified the presence of pes anserinus bursitis and patellar tendinitis in some of our patients. Very few other authors described the presence of periarticular changes including pes anserinus bursitis and patellar tendinitis in people with symptomatic KOA. However, we consider the relevance of assessing these periarticular structures since USG can be a useful tool for the evaluation of superficial bursitis in the knee^32^. Compared with MRI, USG shows a sensitivity of 86.67%, specificity of 100% and k-value of 0.91 for bursitis and 71.4%, specificity of 100% and k-value of 0.82 for suprapatellar bursitis^32^. Actually, we found a higher frequency of pes anserinus bursitis (43.30%) compared with Baker’s cysts (29.38%), a result not so often described in the literature. Future studies should demonstrate whether these structures should also be a target for specific interventions.

Periarticular lesions are quite common in patients with chronic knee pain detected on MRI^33^. In fact, iliotibial band syndrome, semimembranosus-tibial collateral ligament bursitis, anserine bursitis, patellar bursitis and tibiofibular cysts are common features. We fully agree with Hill et al. that periarticular lesions may contribute to knee pain and should be included in the differential diagnosis^33^. It is important to recall that the indication for a knee replacement is pain and not knee pathology.

Many studies have tried to correlate meniscal protrusion to pain and functionality^10,34,35^. However, to assess this structure, we suggest that MRI is the preferred imaging methodology, for its higher resolution. The higher costs involved in MRI prescription and the lower accessibility may restrain the use of MRI. And for this reason, we decided not to systematically access the meniscus using USG, because MRI is a better imaging tool for this diagnosis.

In the present study, higher K&L grades were correlated to a higher number of USG findings, but specifically comparing patients with extreme grades (I and IV, I and III, II and IV). Maybe this correspondence, between the lower number of USG findings and lower K&L grades, as well as a higher number of USG findings and higher K&L grades, could also extend to allow differentiation between intermediate K&L grades and the number of USG findings if the studied population were larger. Although this statistic correlation covered mostly boundaries grades, this may suggest that USG has a correspondent result to X-ray and, therefore, a reliable diagnostic potential compared to the current approach.

Our findings were also similar to previous reports in the literature concerning the influence of the USG changes related to functioning assessments^15,36^. Abd El Monaem et al*.*^15^ described a significant positive correlation between WOMAC and USG-measured joint effusion volume and Baker’s cyst volume. Razek et al*.*^36^ reported that higher WOMAC scores were associated with the presence of joint effusion. In our study, both TUG and WOMAC pain and difficulties to perform exercises scores statistically correlated with the number of USG findings.

Despite the importance of unraveling the association of USG features with pain intensity, K&L scores and functionality in KOA patients, some of these findings may also have a role as predictors of disease progression^29,37^. USG knee effusion was a predictor of joint replacement; being higher or equal to 4 mm versus lower than 4 mm, with a hazard ratio of 2.63; confidence interval 95% 1.70 to 4.06, p < 0.0001^37^. The presence of Baker´s cyst, visualized in 29.38% of our patients, seems to be associated with both clinical (odds ratio (OR) 3.07 (IC95% 1.21 to 7.78) and radiological (OR 2.84, IC95% 1.17 to 6.90) progression in KOA after 2 years of follow-up^29^. This one is a very interesting finding, as the presence of Baker´s cysts can be easily identified by an USG evaluation, without the need for MRI.

Our study has a limitation. The absence of a control group may have limited our findings and conclusions.

To our knowledge, systematic description of these specific USG findings is limited in literature but still important, since osteoarthritis pathogenesis is not totally clear and there is a lack of disease-modifying interventions. Here, the positive association found between periarticular structures and pain questions the current clinical management because it suggests that USG is more sensitive in KOA evaluation, considering it shows changes in most patients even when these are not present in radiographic findings. Accordingly, future studies are needed to clarify if this positive association is result of interference or causality between these two variants, or if they are simply risk markers.

## Conclusion

The main USG findings were joint effusion, pes anserinus bursitis, popliteal cysts and patellar tendinitis. The number of USG findings was associated with VAS, K&L I and III, I and IV, II and IV grades, TUG and pain and difficulty to perform WOMAC scores and should be explored in patients with KOA as potential sources of pain and disability.

## Data Availability

The datasets used and/or analysed during the current study are available from the corresponding author on reasonable request.

## Abbreviations

KOA: Knee osteoarthritis
USG: Ultrasonography
K&L: Kellgren & Lawrence
VAS: Visual analogue scale
TUG: Timed up and go test
WOMAC: Western Ontario and McMaster Universities Osteoarthritis Index
ANOVA: Analysis of variance
BMI: Body mass index
MRI: Magnetic resonance imaging
